# Essay on Medical Pneumatology

**Published:** 1890-02

**Authors:** 


ESSAY ON MEDICAL PNEUMATOLOGY; A Physiological, Clinical, and Therapeutic Investigation of the Gases. By J. N. Demarquay, Surgeon to the Municipal Hospital, etc., Paris. Translated, with Notes, Additions, and Omissions, by Samuel S. Wallian, A. M., M. D., etc. Philadelphia and London: F. A. Davis, Publisher.

This volume contains a "study of the gases of the blood in their physiological condition;" a "Medical history of oxygen and its physiological action;" a chapter on "The preparation and administration of oxygen;" one on the "Therapeutic action of oxygen," and another on "Nitrogen, monoxide of nitrogen, and hydrogen." It covers the entire field of the present knowledge of the various gases, and we, as followers of Hahnemann, may get from its pages a superficial pathogenesis of them. Oxygen is more largely used therapeutically, than any of the other gases. It, no doubt, is a valuable agent, but its true place can only be found after careful proving. It is not applicable to any and all diseases of the respiratory organs, as is claimed by some, but like all other remedial agents it will be useful only in the cases in which it is indicated homœopathically.

